# Hamstring graft size for anterior cruciate ligament repair in a sample of Iraqi men

**DOI:** 10.25122/jml-2023-0071

**Published:** 2023-10

**Authors:** Iskandar Mahdi Alardi

**Affiliations:** 1 Department of Surgery, College of Medicine, University of Al-Qadisiyah, Al Diwaniyah, Iraq

**Keywords:** hamstring graft, anterior cruciate ligament, ligament anterior cruciate, ACL: Anterior Cruciate Ligament

## Abstract

The goal of the current investigation was to measure the size of the anterior cruciate ligament (ACL) graft following hamstring (gracilis and semitendinosus) tendon autograft procedures in a relatively large sample size of Iraqi men. In this study, the quadruple hamstring tendon (semitendinosus and gracilis) was measured in a sample of Iraqi adult males. The study enrolled 300 Iraqi men who underwent anterior cruciate ligament repair at Diwaniyah Teaching Hospital and Alforat Alawast Private Hospital between January 2020 and December 2021. The repair procedure was based on the utilization of the quadruple (gracilis and semitendinosus) hamstring tendon. The primary outcome measure was the size of the tendon, determined using a sizing tube calibrated to 0.5 mm. Out of the sample, 80% of patients had a graft diameter of 7.5 mm, while 15% had a graft diameter of 8 mm, and 5% had a diameter of 7 mm. Body mass index was a poor predictor of graft size, whereas patient height was an excellent predictor of graft diameter. The graft diameter in most Iraqi subjects with anterior cruciate ligament injuries needs to be five bands or even six bands; otherwise, other graft sources like bone-patellar-bone or peroneus longus tendon grafts should be considered. The graft diameter may be reliably anticipated based on the patient’s height.

## INTRODUCTION

Hamstring tendon autografts continue to be a common graft option for the restoration of the anterior cruciate ligament (ACL). These autografts have several advantages over bone-patellar tendon-bone autografts, including reduced postoperative knee pain and a generally easier recovery following surgery. This remains valid despite the availability of various allograft and autograft options for ACL reconstruction. The use of the semitendinosus tendon in the pioneering report of hamstring autograft for anterior cruciate ligament restoration dates back to 1934 [1, 2]. With the semitendinosus and gracilis tendons woven together into a four-stranded construct, two hamstring tendons are now used for ACL autograft restoration. There are over 200,000 cases in the United States of ACL repairs that are carried out each year [3, 4], with patellar tendon autograft and hamstring autograft being the two most popular options [5, 6]. Although every autograft option for ACL reconstruction has benefits and drawbacks, hamstring tendon grafts, in particular, have advantages over patellar tendon autograft, including decreased morbidity of the donor site, pain of the anterior knee, loss and extension, and post-surgical patellofemoral crepitation in addition to skin incisions that are small at the site of the harvest [7]. Despite this, compared to ACL restoration with patellar tendon grafts, several investigations have revealed functional weakness of the hamstring and greater laxity of the joint [8]. There is currently inadequate data to determine whether the type of graft leads to better long-term functional outcomes after ACL restoration [1]. In our country, the utilization of the quadruple hamstring tendon graft is by far the most prevalent kind of grafting in dealing with ACL injury repair. The quadruple hamstring graft is considered stiffer than the patellar bone graft. According to recent reports, the size of the hamstring graft remains the key feature for successful ACL repair surgery [9]. Indeed, the risk of surgery failure and revision rate increases when the ligament reconstruction size is smaller than 8 mm. In the present study conducted in Iraq, the patient sample was selected without consideration for weight, height, or body mass index (BMI). Prediction of the hamstring quadruple graft diameter can be useful in preoperative discussions, offering insight into the choice between similar grafts like bone-tendon-bone (BTB), peroneus longus tendon autograft, or even allograft options. Furthermore, surgeons may explore strategies to increase graft diameter, such as implementing five or six bands. The goal of the current investigation was to assess the anterior cruciate ligament reconstruction length by measuring the size of the graft in a relatively large sample of Iraqi men who underwent hamstring (gracilis and semitendinosus) tendon grafts for ACL injury.

## MATERIAL AND METHODS

In this cross-sectional study, a total of 300 Iraqi men undergoing anterior cruciate ligament repair were enrolled at Diwaniyah Teaching Hospital and Alforat Alawast Private Hospital between January 2020 and December 2021. The repair procedure was based on the use of the quadruple (gracilis and semitendinosus) hamstring tendon graft. The primary outcome was the size of this tendon measured by a sizing tube calibrated to 0.5 mm. The height and weight of patients were recorded by the researcher, and the body mass index was estimated based on the following equation: weight (kg) divided by height (meters) [2]. The height was measured by a stadiometer. During the surgical procedure, an anterior longitudinal incision of approximately 2 cm was made to expose and release the tendon, which was subsequently extracted. The hamstring graft was harvested by a closed tendon stripper. First, the gracilis tendon was harvested, followed by the same procedure for the semitendinosus tendon. After harvesting the graft, the tendon was cleared of all muscle attachments. The two tendons of the graft were then whip-stitched together using a non-absorbable high-strength suture of size two. Subsequently, both tendons were passed through an adjustable end button loop, and the size of the quadruple hamstring graft, composed of four strands, was measured using a graft sizer. The length of the graft (four strands) typically exceeded 10 mm. Data analysis was conducted using the Statistical Package for the Social Sciences (SPSS) software, version 16. Qualitative data were presented as counts and proportions, while quantitative data were summarized using minimum, maximum, standard deviation, and mean values. The Pearson correlation test was employed to explore relationships between graft length and other variables examined in the study. The significance level was considered at a p-value of 0.05 or less.

## RESULTS

The general characteristics of the study participants are presented in Table 1. Table 2 provides insight into the mean length of the quadruple hamstring tendon graft and the distribution of enrolled patients based on graft size. The correlations between ACL-R and anthropometric measurements of the participants are outlined in Table 3. The graft size measured 7.5 mm in 80% of cases, 8 mm in 15%, and 7 mm in 5% of cases. The analysis revealed a significant positive correlation between ACL-R and height (r=0.683, p<0.001) (increased graft size). Age, weight, and BMI were not significant indicators (p<0.05). The Kolmogorov-Smirnov test of normality was employed to assess the distribution of quantitative variables.

**Table 1:** General features of subjects

Characteristics	Result
Number of cases	300
**Gender**	
Males	300 (100%)
**Age (years)**	
Mean ± SD	27.09±7.01
Range	19-35
**Height (meter)**	
Mean ± SD	1.77±0.09
Range	1.63-1.95
**Weight (kg)**	
Mean ± SD	71.82±7.03
Range	68-87
**BMI (kg/m^2^)**	
Mean ± SD	23.28±7.92
Range	19.51-29.38

SD: standard deviation; BMI: body mass index

**Table 2:** The mean length of the quadruple hamstring tendon graft and frequency distribution of patients enrolled

Characteristics	Result
ACL-R (mm)	
Range	7-8”
Mean ± SD	7.56±0.13
7 mm, n (%)	15 (5%)
7.5 mm, n (%)	240 (80%)
8 mm, n (%)	45 (15%)

SD: standard deviation

**Table 3:** Correlation of ACL-R to anthropometric measurements of enrolled patients

Characteristic	r	p-value
Age	0.134	0.201
Height	0.683	<0.001***
Weight	0.128	0.286
BMI	0.204	0.102

## DISCUSSION

The principal findings of the present work can be outlined in two main key points. Firstly, most of our patients had a graft size of less than 8 mm. Secondly, the height of the patients was the main predictor of the graft diameter. The high frequency of ACL injuries has generated a lot of interest in ACL reconstruction methods. However, individual tendon diameter variations and inadequate tendon diameter grafts could lead to graft fragility, inefficiency, and even failure [10-12]. Therefore, to avoid such consequences, the current investigation was conducted over two years, focusing on exploring the association between anthropometric characteristics such as height, BMI, and weight and the size of the hamstring tendon in 300 male individuals who underwent surgical ACL repair. The average age of the participants was 27.09±7.01 years. In a previous study, there was no significant association between the age of the patient and the length of the graft [13]. Similarly, in another study, no significant variations between hamstring tendon size and age intervals were found [14]. The findings of the current study contribute to the growing body of evidence suggesting that there are no appreciable variations in hamstring tendon diameter with age. Previous studies have examined the association between gender and graft size [14, 15]; however, because of the limitation of our study to the male gender, such an association could not be evaluated. Earlier studies have demonstrated a positive and significant correlation between patient height and graft size [13-15], and our results are consistent with these observations. In contrast, our observations do not concur with the conclusions of previous authors [13, 14], who suggested weight or BMI as potential predictors for graft length [13, 14]. Our findings in this regard align with the results reported by other researchers [12]. Orthopedic surgeons are concerned about two main adverse outcomes: graft failure and knee instability [13]. Orthopedists can benefit greatly from the capacity to anticipate tendon diameter based on anthropometric factors because graft diameter has a significant impact on these unexpected outcomes [16]. The hamstring graft has many advantages over other grafts, such as BTB grafts, as it is considered more stable. Another important factor favoring the use of hamstring grafts is that most of our people kneel during praying, which can cause severe pain after BTB. The small size of the graft could increase the failure rate and lead to revision, particularly if the graft's diameter measures less than 8 mm. In instances where the graft's diameter falls below this threshold, it becomes essential to consider alternative graft options or increase the size of hamstring grafts by employing a 5-band or 6-band configuration. As previously mentioned, other graft alternatives are not desirable or available. Western studies have identified the average graft size ranging from 7.9 mm to 8.6 mm. In the Asian population, this dimension averages around 7.9 mm. The results showed that in most patients, the graft size was less than 8 mm, so alternative graft sources should be considered, such as adopting a quadruple graft (5-band) technique achieved by segmenting the semitendinosus tendon into three bands. In our country, the main source of graft is the hamstring tendons. The majority of the Iraqi population had an increased risk of graft failure when using a 4-strand graft due to its diameter falling below 8 mm. This finding has led us to recommend that orthopedic practitioners consider acquiring the skills to perform 5 or 6-strand grafts, which could result in improved outcomes in their surgeries. The main limitation of this study is that the graft length was not considered despite its significant role in influencing graft thickness.

## CONCLUSION

In Iraq, the majority of ACL reconstruction grafts necessitate the utilization of 5-band or 6-band techniques, underscoring the importance of acquiring proficiency in these methods. Exploring alternative graft sources such as peroneus longus tendons or BTB grafts should also be considered. The height of the patient is a reliable predictor of graft size.
